# Molecular characterization of a defensin gene from a hard tick, *Dermacentor silvarum*

**DOI:** 10.1186/s13071-014-0625-0

**Published:** 2015-01-15

**Authors:** Juanjuan Wang, Gang Bian, Wen Pan, Tingting Feng, Jianfeng Dai

**Affiliations:** Institute of Biology and Medical Sciences, Jiangsu Key Laboratory of Infection and Immunity, Soochow University, Suzhou City, Jiangsu Province People’s Republic of China; Wuxi Medical School, Jiangnan University, Wuxi City, Jiangsu Province People’s Republic of China; Soochow University, Building 703, 199 Ren-ai Road, Suzhou, 215123 P.R. China

**Keywords:** Defensin, Antimicrobial peptide (AMP), Tick, *Dermacentor silvarum*

## Abstract

**Background:**

Ticks are distributed worldwide and considered as vectors of many human diseases. Tick defensins, a family of antimicrobial peptides, form the first line of defense against pathogens.

**Findings:**

A defensin-like gene, named Ds-defensin, was identified from a cDNA library of the hard tick *Dermacentor silvarum* collected from northeast China. The full-length cDNA of Ds-defensin was 225 bp, encoding a 74 amino acid peptide. The nucleotide sequence of Ds-defensin shared 98.2% similarity to putative defensin from *Dermacentor marginatus*. RT-PCR results suggested that Ds-defensin was extensively expressed in tick salivary gland and midgut, with a higher expression level in midgut. Ds-defensin showed broad antimicrobial activity against various Gram-positive and Gram-negative bacteria, as well as the fungus *Candida albicans.*

**Conclusions:**

We characterized a functional defensin from *D. silvarum* of China. Ds-defensin showed bactericidal activity against various Gram-positive and Gram-negative bacteria. Ds-defensin can be expected to be introduced to the medical field as a new molecule with antibacterial activity.

**Electronic supplementary material:**

The online version of this article (doi:10.1186/s13071-014-0625-0) contains supplementary material, which is available to authorized users.

## Findings

Ticks, important medical arthropods, have great effects on animal and human health by transmitting various pathogens worldwide. Tick-borne pathogens include viruses, spirochetes, rickettsia, bacteria, and protozoa. They cause diseases such as tick-borne encephalitis, Crimean-Congo hemorrhagic fever, Lyme disease, Q fever, and Rocky Mountain spotted fever. In recent years, a new series of tick-borne diseases have posed a threat to the survival of mankind [1,2]. Many serological surveys also indicated the existence of populations with a variety of tick-borne infectious disease antibodies [3,4].

Hard ticks feed on mammals for a long time (several days), so that they have many opportunities to encounter microbes. Ticks do not have lymphocytes, thymuses, or antibodies. They rely heavily on antimicrobial peptides (AMPs) to defend against microbes so that they can live harmoniously with microbes [5,6]. AMPs are innate immune molecules that kill pathogenic microbes. Defensin is a well-known AMP in ticks. Defensins have been isolated from many species, including mammals, insects, and plants, and provide initial defense against infectious pathogens [7,8]. Several defensins and their isoforms have been identified in tick species including *Ixodes scapularis*, *Amblyomma americanum*, *Dermacentor variabilis*, *Rhipicephalus microplus, Ornithodoros moubata*, *Ixodes ricinus*, *Amblyomma hebraeum* and *Haemaphysalis longicornis* ticks [9–13]. Tick defensins usually contain six cysteine residues, and are usually expressed in the midgut (MG) after blood feeding or pathogen invasion [14,15]. Antimicrobial activity is primarily directed against Gram-positive bacteria, but some isoforms are also effective against Gram-negative bacteria, protozoa, and yeasts [9,15–17].

In our study, we characterized a defensin gene from a cDNA library of *Dermacentor silvarum*. This hard tick species was collected in the field of Heilongjiang Province, located in northeast China. It has been reported to transmit human pathogens, such as tick-borne encephalitis virus, *Anaplasma*, and *Rickettsia* [18–20].

**Figure 1:**
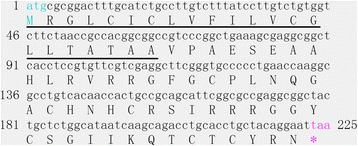
**Nucleotide and deduced amino acid sequences of Ds-defensin from** ***Dermacentor silvarum*** **.** Putative signal peptide of Ds-defensin, as predicted by SignalP 4.1 server, is underlined.

By sequencing a cDNA library of *D. silvarum*, a cDNA clone encoding the precursor of a putative defensin was obtained and named Ds-defensin. The cDNA sequence and deduced amino acid sequence of Ds-defensin are shown in Figure 1. Sequence analysis indicated that the Ds-defensin cDNA was 225 bp long, encoding a 74 amino acid peptide. The predicted protein contained a putative signal peptide cleavage site at amino acid positions 22 to 23, as analyzed by SignalP4.1 software (http://www.cbs.dtu.dk/services/SignalP/). The sequence of Ds-defensin cDNA has been deposited in GenBank with accession number KJ885301.

**Figure 2:**
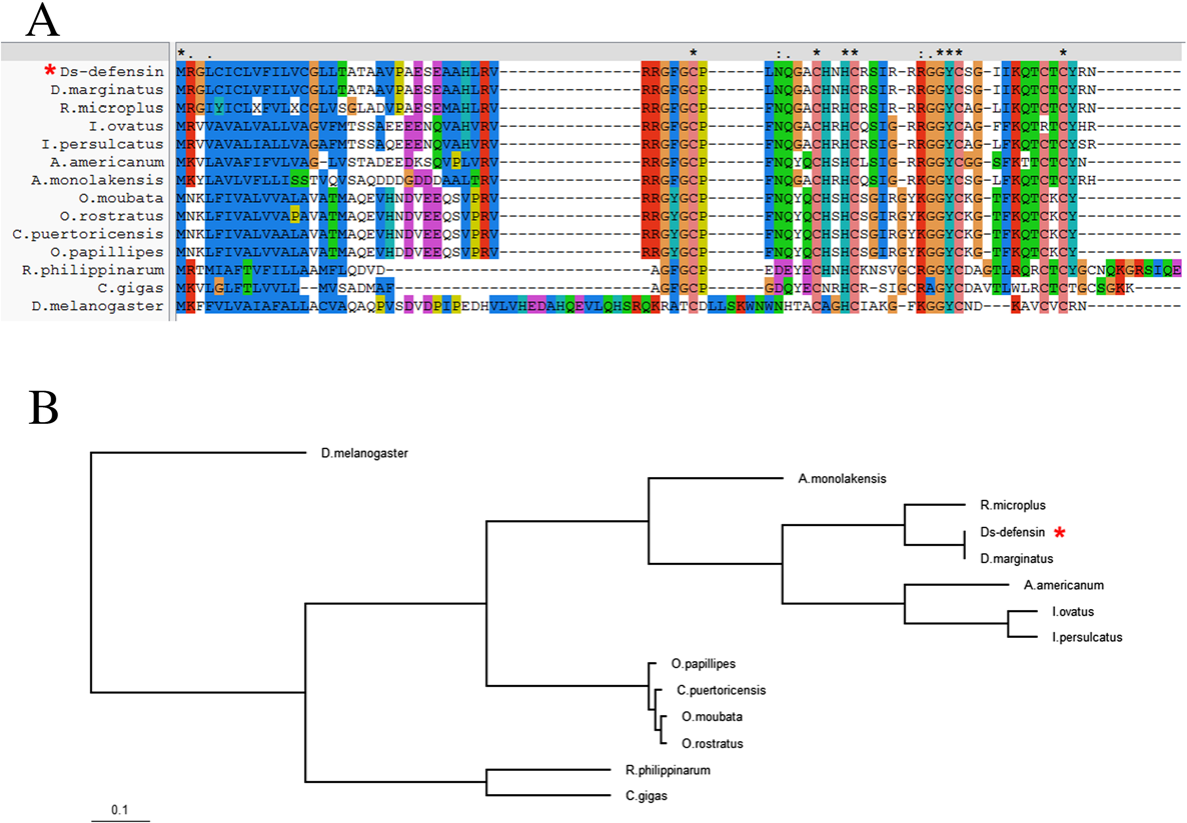
**Sequence alignment (A) and phylogenetic tree (B) constructed with sequences of putative defensins from** ***Dermacentor silvarum*** **and other species.** Ds-defensin is indicated with a red asterisk. Sequence GenBank accession numbers: *Rhipicephalus microplus* [AAO48943], *Ornithodoros moubata* [BAB41028], *Ornithodoros papillipes* [ACJ04425.1], *Ornithodoros rostratus* [ACJ04428.1], *Carios puertoricensis* [ACJ04429.1], *Argas monolakensis* [ABI52766.1], *Dermacentor marginatus* [ACJ04433.1], *Ixodes ovatus* [BAH09305.1], *Ixodes persulcatus* [BAH09304.1], *Amblyomma americanum* [ABI74752.1], *Ruditapes philippinarum* [ADO32580.1], *Crassostrea gigas* [ACQ76287.1], and *Drosophila melanogaster* [AAF58855.1].

To identify the evolutionary relationships between Ds-defensin and other defensin-like genes in invertebrate species, sequence alignment and phylogenetic analysis were conducted using multiple sequence alignment software Clustal X2 (http://www.clustal.org/). The nucleotide sequence of Ds-defensin shared 98.2% identity with a putative defensin from *Dermacentor marginatus*, and their amino acid sequences were 100% identical, suggesting that these two tick species were closely related (Figure 2). Sequence alignment results suggest that Ds-defensin shared high similarity to putative defensins from hard tick species, such as *R. microplus* (79.73%), *I. ovatus* (58.11%), *I. persulcatus* (59.46%), and *A. americanum* (55.56%), and low similarity to putative defensins from soft ticks, such as *O. moubata* (43.84%), *O. papillipes* (43.84%), *O. rostratus* (43.84%), and *C. puertoricensis* (43.84%). All these defensins contained six conserved cysteine residues, including defensins from fruit fly (*Drosophila melanogaster*) or marine mollusk species (*Crassostrea gigas* and *Ruditapes philippinarum*) (Figure 2A). Phylogenetic tree analysis also suggested that these putative defensin sequences fell into three distinct clusters. One cluster comprised defensins from hard ticks (*D. silvarum*, *D. marginatus*, *R. microplus*, *A. monolakensis*, *A. americanum*, *I. ovatus*, and *I. persulcatus*). Another cluster comprised defensins from soft ticks (*O. moubata*, *O. papillipes*, *O. rostratus*, and *C. puertoricensis*), and the other cluster comprised defensins from marine mollusk species (*A. americanum* and *R. philippinarum*) (Figure 2B). Although defensins were evolutionarily conserved at the critical amino acid positions (six cysteine residues), they also exhibited significant changes during evolution.

**Figure 3:**
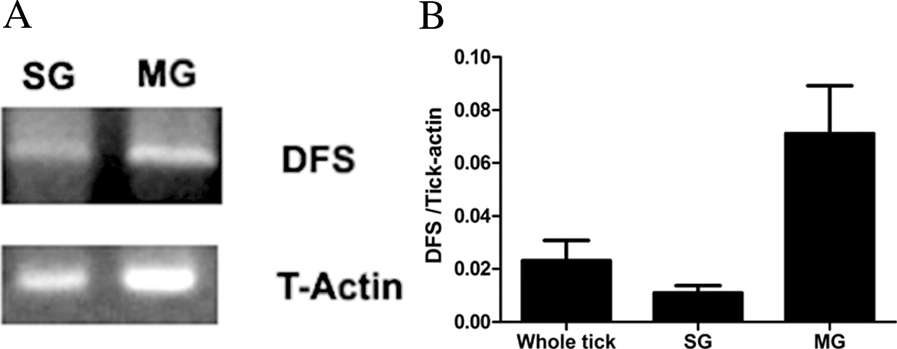
**Expression of Ds-defensin mRNA in ticks. (A)** Expression of Ds-defensin in tick salivary gland (SG) and midgut (MG) as determined by RT-PCR. Tick actin gene was amplified as the loading control. **(B)** Relative expression level of Ds-defensin in SG, MG, and whole ticks, as determined by quantitative RT-PCR. The copy number of Ds-defensin was normalized to tick actin. Results are expressed as the mean + SEM. Results represent at least three independent experiments.

To investigate the mRNA expression of Ds-defensin in different tissues of *D. silvarum*, RNA was extracted from whole ticks, salivary gland (SG), and midgut (MG). Transcript expression analysis of the Ds-defensin gene was conducted by RT-PCR using Ds-defensin gene-specific primers. As shown in Figure 3, Ds-defensin was extensively expressed in MG and SG, and the expression level in MG was significantly higher than that in SG or whole ticks (Figure 3B). These data were consistent with previous findings, and suggest that Ds-defensin may play important roles in protecting ticks against microorganisms in MG and SG.

To test the antimicrobial activity of Ds-defensin, the mature form of Ds-defensin (a 52 amino acid peptide) was synthesized and purified by high-performance liquid chromatography (GL Biochem, Shanghai, China). The peptide was dissolved in PBS buffer containing 0.05% Tween 20 and 1 μM β-merkaptoethanol (at a stock concentration of 1000 μM), and diluted properly when used in the antimicrobial assay. The target bacteria used in the bactericidal assay were purchased from China Veterinary Culture Collection Center (CVCC). Four Gram-positive bacteria, namely, *Staphylococcus aureus* (CMCC26003), *Bacillus pumilus* (CMCC63202), *Micrococcus luteus* (CMCC63202), and *Mycobacterium bovis* (carbenicillin-resistant strain); and three Gram-negative bacteria, namely, *Salmonella typhimurium* (CVCC542), *Pseudomonas aeruginosa* (CVCC2000), and *Escherichia coli* (CMCC44103), were used in this study. Microbial strains were grown to an OD_600nm_ of 0.4–0.6 at 37°C in Poor Broth media (1% w/v tryptone and 1% w/v NaCl) (except for *M. bovis*, which was grown in Middlebrook 7H9 Broth (BD-Difco, USA) with carbenicillin). Approximately 90 μL of inocula of microbial strains (diluted with PB media to an OD_600nm_ of 0.01) and 10 μL of various concentrations of Ds-defensin (0.1–50 μM) were added to the well of a 96-well plate. The mixture was grown for 20 h (for *M. bovi,* incubated for 48 h) at 37°C and 250 rpm. Antimicrobial activity was evaluated by measuring the absorbance of the bacterial culture at OD_600nm_. As shown in Table 1, Ds-defensin showed significant antimicrobial activities against Gram-positive bacteria with a minimal inhibitory concentration (MIC) less than 50 μM. For *B. pumilus*, Ds-defensin showed potent antimicrobial activities at 1 μM. Ds-defensin was less effective to Gram-negative bacteria. The MIC values to *S. typhimurium* and *P. aeruginosa* were greater than 50 μM. Ds-defensin did not inhibit the growth of *E. coli*, but it significantly inhibited the growth of antibiotic-resistant *M. bovis* (MIC = 20 μM). To test whether Ds-defensin also influences the growth of fungi, *Candida albicans* (CAU0037) was used in the antimicrobial assay. *C. albicans* replication was significantly inhibited when the concentration of Ds-defensin exceeded 10 μM in Modified Martin Broth (Solarbio, Beijing, China), but the MIC to *C. albicans* was greater than 50 μM (Table 1). As shown by hemolytic assay, Ds-defensin was harmless to human erythrocytes, in concentrations of up to 20 μM (Additional file 1: Figure S1, A). Furthermore, Ds-defensin showed no detectable cytotoxicity to mammalian cell lines (Additional file 1: Figure S1, B), and did not infect the replication of vesicular stomatitis virus (VSV) in 293 T cells (data not shown).

**Table 1:** **Antimicrobial activity of Ds-defensin**

**Strain of microbes**	**MIC (μM)**
**Gram-positive bacteria**	
*Bacillus pumilus (CMCC63202)*	1
*Staphylococcus aureus (CMCC26003)*	20
*Micrococcus luteus (CMCC63202)*	50
*Mycobacterium bovis (carbenicillin-resistant)*	20
**Gram-negative bacteria**	
*Salmonella typhimurium* (CVCC542)	>50
*Pseudomonas aeruginosa* (CVCC2000)	>50
*Escherichia coli (CMCC44103)*	no effect
**Fungus**	
*Candida albicans (CAU0037)*	>50

MIC: minimal peptide concentration required for total inhibition of cell growth in liquid medium. Determination of MICs was performed at least three times in triplicates.

Defensins are present in all types of organisms from humans and plants to arthropods. Defensins are AMPs that form the first line of defense against pathogens. A common function of defensins from all organisms is to lyse bacterial cells; however, the amino acid sequences of defensins show high diversity. Sequence analysis showed that the Ds-defensin peptide had 98.2% identity at the nucleotide level and 100% identity at the amino acid level to a putative defensin from *D. marginatus*, another hard tick species. Our data also show that defensins from hard and soft ticks shared a high degree of variability. The differentiation could be influenced by a diverse strategy of blood intake and types of pathogens that each tick species has encountered during their evolutionary history and geographical isolation [21].

The incidence of tick-borne diseases has steadily increased over the past few years, and effective vaccines against most tick-borne pathogens are currently unavailable [22]. Defensin is an AMP that is not yet affected by antibiotic-resistance mechanisms [23]. Defensins have been identified in both soft and hard ticks, and they show different profiles of antimicrobial activity. For example, Chrudimská and colleagues characterized two defensin isoforms (Def1 and Def2) from the hard tick *I. ricinus*, and showed that both Def1 and Def2 have significant bactericidal effects on Gram-positive bacteria but they are insensitive to Gram-negative bacteria, yeasts, and viruses [24]. The same group recently characterized another defensin (defDM) from the hard tick *D. marginatus*, and suggested that defDM has inhibitory effects on Gram-positive bacteria and a popular tick-borne pathogen *Borrelia afzelii* [25]. Lu *et al.* reported that a defensin-like peptide (DLP) from hard tick *H. longicornis* has potent antimicrobial activities against bacteria and fungi (*C. albicans*), and even shows strong antimicrobial ability against drug-resistant microorganisms [6]. A recent review by Wang *et al.* summarized that the hard tick *I. scapularis* has two multigene families of DLPs [26]. The core region of *I. scapularis* defensin scapularisin-20 exhibits a wide spectrum of antimicrobial activity against both Gram-positive and Gram-negative bacteria, with higher potency to Gram-positive bacteria than to Gram-negative bacteria. The novel defensin we identified from the hard tick *D. silvarum* also has a distinct antimicrobial profile to different microbes. Ds-defensin showed strong potency against some Gram-positive bacteria (*S. aureus*, *B. pumilus*, *M. luteus*, and *M. bovis*), but had less effect on Gram-negative bacteria (*S. typhimurium*, *P. aeruginosa*, and *E. coli*). At a dose higher than 10 μM, Ds-defensin showed significant antifungal activity against *C. albicans* growth and could inhibit the antibiotic-resistant strain of *M. bovis*. Ds-defensin was not hemolytic at 20 μM, and had no detectable cytotoxicity against various human cells. These data suggest that Ds-defensin could be safely used in mammalian systems as a potential antimicrobial reagent against various Gram-positive bacteria and some fungi.

## Additional file

Additional file 1: Figure S1. Hemolytic and cytotoxic assay of Ds-defensin. (A) Hemolytic effect of Ds-defensin to mouse erythrocytes. Hemolytic activity was measured by co-incubating 2% (vol/vol) erythrocyte suspension with various concentration of Ds-defensin for 2 hours at 37°C. (B) Ds-defensin had no cytotoxic effect on mammalian cell lines. Ds-defensin was added into the cell media at a final concentration of 10 μM and incubated with various cells for 24 h. The cell viabilities were measured using a CellTiter-Glo kit (Promega, USA).
