# Supplementary figures and images for: Molecular characterization of a defensin gene from a hard tick, Dermacentor silvarum
Source: Parasit Vectors. 2015 Jan 15;8:25. doi: 10.1186/s13071-014-0625-0 (PMC4311433; doi:10.1186/s13071-014-0625-0)

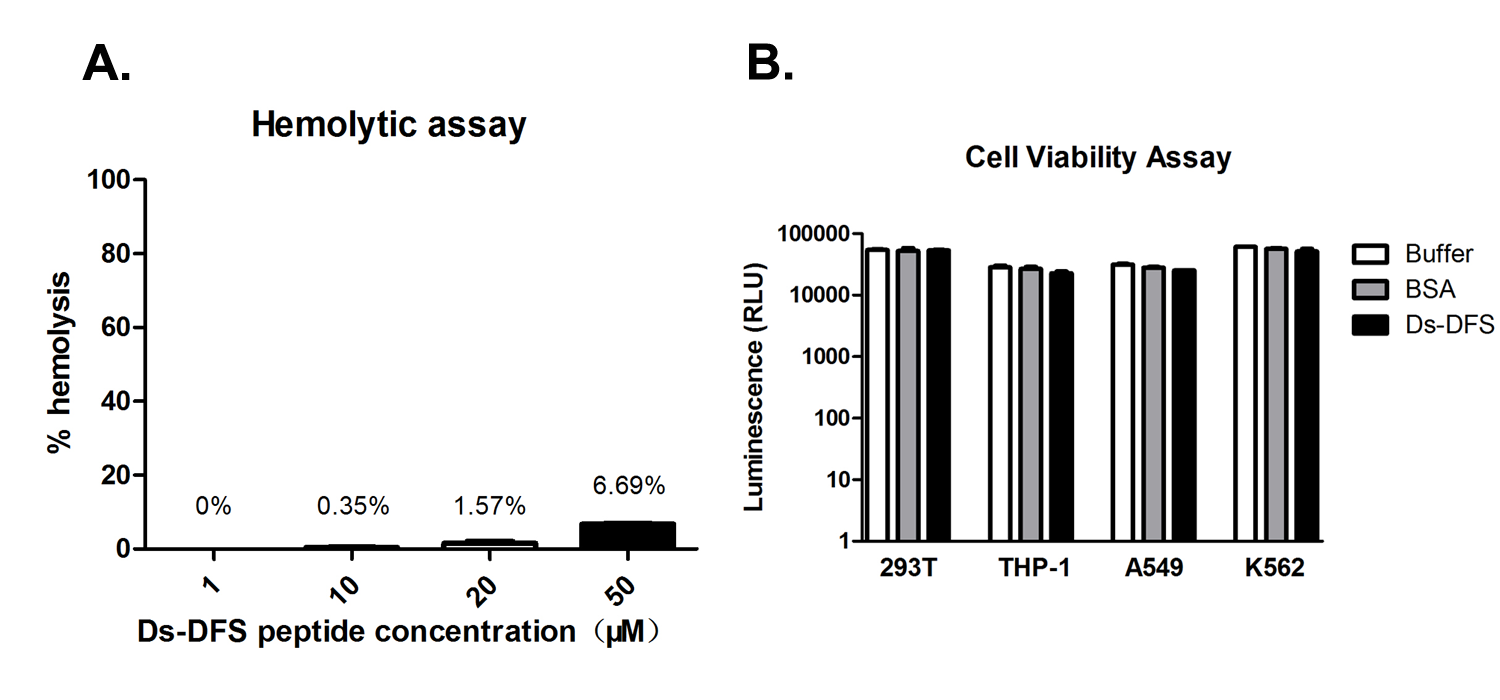

Supplement: Additional file 1: Figure S1. — Hemolytic and cytotoxic assay of Ds-defensin. (A) Hemolytic effect of Ds-defensin to mouse erythrocytes. Hemolytic activity was measured by co-incubating 2% (vol/vol) erythrocyte suspension with various concentration of Ds-defensin for 2 hours at 37°C. (B) Ds-defensin had no cytotoxic effect on mammalian cell lines. Ds-defensin was added into the cell media at a final concentration of 10 μM and incubated with various cells for 24 h. The cell viabilities were measured using a CellTiter-Glo kit (Promega, USA). [file 13071_2014_625_MOESM1_ESM.tiff]
